# Informed consent for clinical treatment in low-income setting: evaluating the relationship between satisfying consent and extent of recall of consent information

**DOI:** 10.1186/s12910-017-0227-4

**Published:** 2017-12-02

**Authors:** Ikenna I. Nnabugwu, Fredrick O. Ugwumba, Emeka I. Udeh, Solomon K. Anyimba, Oyiogu F. Ozoemena

**Affiliations:** 1 Department of Surgery, College of Medicine, University of Nigeria, Enugu Campus P M B, State, Enugu, 01129 Nigeria; 2 University of Nigeria Teaching Hospital, Ituku-Ozalla, Enugu, Nigeria

**Keywords:** Informed consent, Information recall, Patients’ concerns

## Abstract

**Background:**

Treatment informed consent aims to preserve the autonomy of patients in the clinician – patient relationship so as to ensure valid consent. An acceptable method of evaluating understanding of consent information is by assessing the extent of recall by patients of the pieces information believed to have been passed across. When concerns are not satisfactorily addressed from the patients’ perspective, recall of consent information may be low.

**Methods:**

This study is a questionnaire – based cross – sectional interview of consecutive adult surgical patients who could give their respective medical histories and who were booked for elective major surgical procedures over a period of 7 months in a tertiary health institution in southeastern Nigeria. Four to five days after a formal consent session, during ward admission, extent of recall of information on the nature of the disease condition or diagnosis, the nature of the planned procedure and the risks involved in the planned procedure were assessed and analyzed on the background of how satisfying the consent sessions were from individual patient’s perspective.

**Results:**

Generally, the recall of nature of disease condition and nature of planned procedure is better than recall of risks involved in the planned procedure. More specifically however, recall in these 3 domains is significantly better among the patients that affirmed that their concerns were satisfactorily addressed.

**Conclusion:**

The findings from this study support that no effort should be spared in ensuring that the consent information are satisfying to the patients from the patients’ viewpoint.

**Electronic supplementary material:**

The online version of this article (10.1186/s12910-017-0227-4) contains supplementary material, which is available to authorized users.

## Background

In modern clinical practice, there is a conscious effort by health care teams to carry patients along in all decisions concerning the latter’s medical care. Over the years, the principal-agent relationship in healthcare services has undergone some modifications through the concept of consent with a shift from ‘obtaining consent’ from patients to educating patients to ‘give informed consent’ prior to any intervention with the aim of improving patients’ satisfaction and quality of care [1]. The concept of informed consent for clinical treatment, an important aspect of biomedical ethics, recognizes the autonomy of patients in the relationship of clinicians and patients. In addition to making the patients understand that their autonomy as individuals is not in any way interfered with, they are made to appreciate that the healthcare teams are acting with good intentions, and all precautions are taken to avoid unintended outcomes [2]. The clinicians demonstrate that they understand the patients’ health challenges and that the available health system is capable in one way or the other of offering solutions to the latter’s suffering, but with the understanding of all concerned that there may be unforeseen events [3]. The process of informed consent for treatment also attempts to bridge the gap in knowledge between the clinicians and the patients, of the patients’ health challenges, of the various options available for treatment, and of the implications of each and every treatment option including the option of declining treatment [4]. If the patient is a minor or is cognitively impaired, a legally accepted proxy or surrogate receives information and gives the informed consent on the patient’s behalf [4, 5].

Several strategies are deployed in achieving the desired goals of giving an informed consent and several sessions may be needed. These strategies include oral discussions, handing out of manuscripts containing needed information to the patients, referring patients to appropriate education materials and websites, and the playback of appropriately structured audio and video recordings [6–8]. The information should be in the language that the patients understand. In effect, from the patients’ perspective, the process of ‘getting informed’ commences at first presentation and continues through the period of the patient – doctor relationship. Each patient formally gives his consent for a treatment option by appending his or her signature on an officially recognized document, the consent form, an action which confirms that a formal session of getting informed took place, but which still leaves the patient retaining the right of withdrawal of consent at any point before the planned intervention.

The extent of comprehension of the consent information and the subsequent recall of this information believed to have been given are related to the extent of interactions with and exposure of the patient to the care-givers in the health care facility [9]. Because there are many sources of information of various sorts to the patients on the nature and the management of their disease conditions, a formal session or sessions are necessary to correct wrong notions, clarify confusing issues, and summarize the necessary information needed by the patient for rational decision-making. In these interactive consent sessions, the patients are given adequate information for effective participation in this shared decision-making process. Valid consent is said to have been actualized where there are physician disclosure of all necessary information and patient capacity to rationally participate, leading to patient understanding of the content of the discussion in a context of voluntariness thereby discouraging making decisions based on paternalism [10]. Information recall by patients, which is an acceptable measure of comprehension, is reasonably high after such valid informed consent sessions [11].

The informed consent process has largely been introduced into clinical practice, but the strategies employed in achieving it vary from society to society. In Nigeria, a lower medium-income country, literacy level of patients is on the average low, belief in the theology of predestination is strong, infrastructural development of the health institutions is mostly rudimentary, and the influence of the extended family system is strong [12, 13]. In addition, the consent forms in most of our institutions are generic with paucity of information required for valid informed consent to be given, [14] and most of the local languages are poorly developed leading to imprecision in delivery of information concerning available healthcare goods and services. In spite of these challenges, it is believed that where they are given adequate information, our patients have the capacity to comprehend thereby allaying all fears and improving patients’ satisfaction. The extent of recall by a patient of the various pieces of information received during the consent sessions for specific treatment modalities [7, 15] may be related to whether the patient found the consent information satisfying or not. So patterning our data collection questionnaire to similar instruments previously developed for the assessment of extent of recall of consent information [6, 16], we designed this study with the aim of evaluating, among our adult elective surgical patients, the recall of the information received during the informed consent process. This study hypothesizes that the extent of recall of consent information by the adult patient is related to the degree to which the patient’s concerns with respect to the planned surgery were addressed.

## Methods

As part of evaluation of each patient for elective surgery in the outpatient clinics, standard operational procedure demands that the surgeon gives to the patient all the information necessary for the latter to give an informed consent. Such information includes, but not limited to, the nature of diagnosis and the nature of the treatment options available with the attendant risks and successes. On the day of admission into the ward for the planned surgical operation, on the premise that all necessary information has been well-received, the consent form is signed by the patient.

This cross sectional survey was conducted in the University of Nigeria Teaching Hospital, Ituku-Ozalla, Enugu, in south-east Nigeria. Approximately 1606 elective surgeries were carried out on patients 18 years and above in the institution’s main theatre complex in the preceding year (2014). Based on this number therefore, we estimated a sample size of 310 using Survey System® software. From February 2015 to August 2015, a total of 401adult patients 18 years and older were scheduled for elective surgeries. Nine patients declined to participate while 23 patients did not meet an inclusion criterion to participate in the study. This inclusion criterion is that the patient must have been able to give his clinical history himself at presentation. The resultant 369 participants were drawn from the Urology, General Surgery and Orthopaedic units, and these gave consent to participate in the study.

Each patient was given procedure-specific consent information verbally by the surgeon as part of care protocol about 4 or 5 days prior to the date of the intended procedure: on an outpatient clinic visit preceding the date of the proposed procedure. Expositions on the nature of the disease condition, the nature of the planned procedure and the risks involved in the procedure form part of the content of each consent session with the objective of addressing all concerns arising from any pre-conceived notions. For the purposes of this study, the study questionnaire was administered to each participating patient on admission into the ward a day prior to the procedure day. The response to the questionnaire revealed the extent of recall of information believed to have been given earlier to the patient, and hence the areas that required ‘top up’ information by the surgeon before the eventual endorsement of the consent form by the patient. The questions in the questionnaire were non-leading and options with respect to answers to the questions were not provided. Four Intern doctors, who scored highest in a quiz after tutelage on the administration of the questionnaire assisted with administering the questionnaires to the various patients, translating and explaining the items therein whenever there was need. There were questions on the nature of the diagnosis, the nature of the planned procedure and the risks involved in the procedure. The response to each of these questions was documented. Where the patient had forgotten what he believed was the answer, ‘forgotten’ was noted; and where he believed he was not given that information during the consent session, ‘uninformed’ was noted. The last question sought to ascertain from the patient (respondent) the extent to which the patient’s concerns during the consent session were addressed by the surgeon, and the response to this particular question was ‘satisfactory’, ‘not satisfactory’ or ‘unsure’. The documented responses were adjudged to be ‘correct’ or ‘incorrect’ by the surgeon during data collation. Patients whose procedures were carried out in the outpatient clinics, wards or emergency theatre complex were not included in the study. The University of Nigeria Health Research Ethics committee gave approval for this study.

For ease of analysis, responses that were judged incorrect’, ‘forgotten’ or ‘uninformed’ were classed as ‘Inappropriate Response’ while responses that were judged ‘correct’ were classed as ‘Appropriate Response’. Similarly, with respect to the last question, ‘Affirmative’ was used for ‘satisfactory’ responses while ‘Negative’ was used for ‘not satisfactory’ and ‘unsure’ responses. The data obtained was analyzed using crosstabs descriptive analyses of SPSS version 20. The figure was produced with MS Excel 2007.

## Results

There are 369 respondents between the ages of 18 years and 82 years (mean 44.1 ± 17.7 years). Among them, 207 (56.1%) are <45 years, 173 (46.9%) are females, and 279 (75.6%) acquired at least a secondary level of formal education. They are mostly Nigerians (99.7%), of the Igbo tribe (91.9%) and residing in the south-eastern region of the country. Table 1 below is a summary of the responses obtained from the questions on nature of diagnosis (Q1), nature of planned procedure (Q2) and risks involved in the planned procedure (Q3).

**Table 1:** Summary of the responses to the questions according to age, gender and highest formal education attained

			Appropriate Response	Inappropriate Response	Total	χ2
Question 1	Age	<45 yrs	169 (81.6%)	38 (18.4%)	207 (100%)	0.10
		≥45 yrs	120 (74.1%)	42 (25.9%)	162 (100%)	
	Gender	Female	153 (88.4%)	20 (11.6%)	173 (100%)	<0.001
		Male	136 (69.4%)	60 (30.6%)	196 (100%)	
	Educational status	≤6 yrs	56 (62.2%)	34 (37.8%)	90 (100%)	<0.001
		>6 yrs	233 (83.5%)	46 (16.5%)	279 (100%)	
Question 2	Age	<45 yrs	133 (64.3%)	74 (35.7%)	207 (100%)	0.83
		≥45 yrs	102 (63.0%)	60 (37.0%)	162 (100%)	
	Gender	Female	121 (69.9%)	52 (30.1%)	173 (100%)	0.01
		Male	114 (58.2%)	82 (41.8%)	196 (100%)	
	Educational status	≤6 yrs	46 (51.1%)	44 (48.9%)	90 (100%)	0.01
		>6 yrs	189 (67.7%)	90 (32.3%)	279 (100%)	
Question 3	Age	<45 yrs	51 (24.6%)	156 (75.4%)	207 (100%)	0.13
		≥45 yrs	29 (17.9%)	133 (82.1%)	162 (100%)	
	Gender	Female	41 (23.7%)	132 (76.3%)	173 (100%)	0.38
		Male	39 (19.9%)	157 (80.1%)	196 (100%)	
	Educational status	≤8 yrs	8 (8.9%)	82 (91.1%)	90 (100%)	0.001
		>8 yrs	72 (25.8%)	207 (74.2%)	279 (100%)	

[Question 1: recall of nature of disease condition; Question 2: recall of nature of planned surgery; Question 3: recall of risks involved in planned surgery; χ2: Fisher exact test; Educational status captures duration of formal education]

**Fig. 1:**
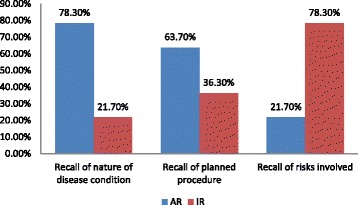
A bar chart showing the proportions of appropriate and inappropriate responses obtained from the respondents in recalling the nature of the disease condition, the planned procedure and the risks involved. In the figure, ‘AR’ means Appropriate Response while ‘IR’ means Inappropriate Response

About 78.3% of the 369 respondents recalled appropriately the nature of the disease condition, 63.7% recalled appropriately the planned surgery, and only 21.7% recalled the risks involved in the planned surgery. This is summarized in the Fig. 1 below.

Responses from respondents who admitted that their concerns were satisfactorily addressed constituting the ‘Affirmative’ group of responses was matched against the ‘Negative’ group of responses from the other respondents who did not admit that their concerns were satisfactorily addressed. Seventy-one percent (71.0%) of those <45 years of age (*n* = 207) and 63.6% of those ≥45 years of age (*n* = 162); 74.0% of females (*n* = 173) and 62.2% of males (*n* = 196); and 50.0% of those with no or primary level of formal education (*n* = 90) and 73.5% of those with post-primary level of formal education (*n* = 279) affirmed that their concerns were satisfactorily addressed. Further analysis was done with respect to the three questions under consideration and the results displayed in Tables 2, 3 and 4.

**Table 2:** Shows the result of matching the ‘Affirmative’ group of responses against the ‘Negative’ group of responses to the question (Q1): may I know your understanding of your disease condition?

			Appropriate Response	Inappropriate Response	Total	χ2
Affirmative	Age	<45 yrs	132 (89.8%)	15 (10.2%)	147 (100%)	0.03
		≥45 yrs	82 (79.6%)	21 (20.4%)	103 (100%)	
	Gender	Female	119 (93.0%)	9 (7.0%)	128 (100%)	0.001
		Male	95 (77.9%)	27 (22.1%)	122 (100%)	
	Educational status	≤6 yrs	31 (68.9%)	14 (31.1%)	45 (100%)	0.002
		>6 yrs	183 (89.3%)	22 (10.7%)	205 (100%)	
Negative	Age	<45 yrs	37 (61.7%)	23 (38.3%)	60 (100%)	0.85
		≥45 yrs	38 (64.4%)	21 (35.6%)	59 (100%)	
	Gender	Female	34 (75.6%)	11 (24.4%)	45 (100%)	0.03
		Male	41 (55.4%)	33 (44.6%)	74 (100%)	
	Educational status	≤8 yrs	25 (55.6%)	20 (44.4%)	45 (100%)	0.24
		>8 yrs	50 (67.6%)	24 (32.4%)	74 (100%)	

[χ2: Fisher exact test; Educational status captures duration of formal education;]

**Table 3:** Summary of the result of matching the ‘Affirmative’ group of responses against the ‘Negative’ group of responses to the question (Q2): may I know your understanding of the planned procedure?

			Appropriate Response	Inappropriate Response	Total	χ2
Affirmative	Age	<45 yrs	115 (78.2%)	32 (21.8%)	147 (100%)	0.45
		≥45 yrs	76 (73.8%)	27 (26.2%)	103 (100%)	
	Gender	Female	102 (79.7%)	26 (20.3%)	128 (100%)	0.24
		Male	89 (73.0%)	33 (27.0%)	122 (100%)	
	Educational status	≤6 yrs	27 (60.0%)	18 (40.0%)	45 (100%)	0.01
		>6 yrs	164 (80.0%)	41 (20.0%)	205 (100%)	
Negative	Age	<45 yrs	18 (30.0%)	42 (70.0%)	60 (100%)	0.13
		≥45 yrs	26 (44.1%)	33 (55.9%)	59 (100%)	
	Gender	Female	19 (42.2%)	26 (57.8%)	45 (100%)	0.43
		Male	25 (33.8%)	49 (66.2%)	74 (100%)	
	Educational status	≤8 yrs	19 (42.2%)	26 (57.8%)	45 (100%)	0.43
		>8 yrs	25 (33.8%)	49 (66.2%)	74 (100%)	

[χ2: Fisher exact test; Educational status captures duration of formal education]

**Table 4:** Shows the result of matching the ‘Affirmative’ group of responses against the ‘Negative’ group of responses to the question (Q3): what are the risks of the planned procedure?

			Appropriate Response	Inappropriate Response	Total	χ2
Affirmative	Age	<45 yrs	45 (30.6%)	102 (69.4%)	147 (100%)	0.32
		≥45 yrs	25 (24.3%)	78 (75.7%)	103 (100%)	
	Gender	Female	36 (28.1%)	92 (71.9%)	128 (100%)	1.00
		Male	34 (27.9%)	88 (72.1%)	122 (100%)	
	Educational status	≤6 yrs	6 (13.3%)	39 (86.7%)	45 (100%)	0.02
		>6 yrs	64 (31.2%)	141 (68.8%)	205 (100%)	
Negative	Age	<45 yrs	6 (10.0%)	54 (90.0%)	60 (100%)	0.74
		≥45 yrs	4 (6.8%)	55 (93.2%)	59 (100%)	
	Gender	Female	5 (11.1%)	40 (88.9%)	45 (100%)	0.50
		Male	5 (6.8%)	69 (93.2%)	74 (100%)	
	Educational status	≤8 yrs	2 (4.4%)	43 (95.6%)	45 (100%)	0.32
		>8 yrs	8 (10.8%)	66 (89.2%)	74 (100%)	

[χ2: Fisher exact test; Educational status captures duration of formal education]

## Discussion

It is standard operative procedure just as it is an ethical principle that patients are given adequate information to allow for rational decision making prior to their signing of the consent form. In doing this, the patients are made to possess adequate knowledge of their health condition and the treatment options available; guided by the ethical principles of autonomy, beneficence, non maleficence and justice [2, 17]. Paternalism in the patient-doctor relationship is thereby discouraged. Interestingly, many studies have documented that patients’ recall of information known to have been made available during informed consent sessions has been generally poor [18, 19]. In instances where the cognitively sound patient is not given the needed information [18] or may not have met the surgeon up until the day of surgery [20], the knowledge base of the patient is usually lower.

Often patients would acknowledge having discussions with their surgeons about their health challenges and treatment modalities. They (patients) are usually able to recollect as well, the chain of referrals including interventions leading to the present point of healthcare [21]. However, recall of specific and important information known to have been delivered during such discussions with surgeons has been recognized repeatedly to be deficient. In the light of this challenge, many strategies have been adopted to increase the extent of recall of the contents of such consent discussions with the proviso that recall is a measure of understanding [6–8]. Understanding expectedly clarifies doubts or concerns.

In all, 78.3% of our respondents could recall appropriately the nature of their disease conditions while 63.7% recalled adequately the nature of the planned procedure (Fig. 1). Mexas et al. in a trial-related consent study recorded 82% correctly answered questions within 24 h of completing the consent process [17]. However, their questions used dichotomous responses unlike ours that were non-leading open-ended in structure. In another study, only 26% of the test group remembered correctly the surgery undergone the day after the surgery [22]. Such a very low recall might not be unconnected to the timing of the administration of the test questions. Interestingly, only 21.7% of the respondents could recall at least one other complication of the planned procedure apart from death, unlike the finding by Chiapponi et al. in Germany [23] and Uzzaman et al. [24] in London where 43.5% and 22.1% of the respondents respectively could not recall any possible complication related to the planned surgery. The reason for the difference is likely to be related to the difference in socio-economic settings.

There is no significant evidence from this study that younger respondents (< 45 years) considered in isolation recall the nature of the disease conditions (*p* = 0.10), the nature of the planned surgeries (*p* = 0.83 or the risks associated with the planned surgeries (*p* = 0.13) more appropriately than older respondents (Table 1); a situation which persists on analysis of the responses of the respondents that could not affirm that their concerns were satisfactorily addressed (Table 2, Table 3 and Table 4). In the study by Mexas et al. [17], age was not found to influence recall, but in the studies by Rosique et al. [25], Crepeau et al. [26] and Sahin et al. [27], younger patients recalled more information than older patients. In this study however, when the responses of patients who affirmed that their concerns were satisfactorily addressed were disaggregated for age, there is strong evidence that recall of nature of disease condition (*p* = 0.03), but not nature of planned surgery (*p* = 0.45) or risks involved in planned surgery (*p* = 0.32) is better in the younger age groups. Our finding may be due to limited access of our younger patients to other sources of appropriate medical information including the internet which leaves both younger and older patients with similar knowledge base.

Comparing in general, the responses obtained from the female respondents to the responses from the male respondents, there is strong evidence (Table 1) that a greater proportion of females produced appropriate recall of the nature of the disease conditions (*p* < 0.001) and the planned surgical procedure (*p* = 0.01), but no evidence with respect to the risks involved in the planned surgical procedure (*p* = 0.38). The reason for this difference is not obvious from this study, but may be related to the observation that 80.9% of the 173 female respondents had at the minimum a post-primary level of formal education as against 70.9% of the 196 male respondents with a post-primary level of formal education. Now, considering the responses from female and male respondents that could not affirm that their concerns were satisfactorily addressed, there are no significant differences in recall in the three domains of interest. However, among the respondents who affirmed, extent of recall of nature of disease condition (*p* = 0.001), but not the nature of planned surgery (*p* = 0.24), or risks involved in the planned surgery (*p* = 1.00) by females appears to be significantly higher.

There is strong evidence from this study that higher level of formal education attained by respondents positively influences the extent of recall of consent information across all domains of interest (Table 1). This is particularly so within the ‘Affirmative’ group where higher level of formal education is associated with higher proportion of appropriate recall (Tables 2, 3, 4). Similar results were obtained by Pathak et al. among women [28] and Dahl et al. among parents of children scheduled for emergency procedures [29]. Appropriate recall of risks of planned surgeries is generally poor at 21.7% of obtained responses. A possible explanation may be that risks are not usually emphasized in many surgeries expected to be generally successful to avoid creating undue anxiety in patients [30, 31]. Alternatively, in an attempt to limit interference with the highlighted treatment option, patients may block off information about risks and alternatives, assimilating little or nothing with respect to such information [32]. In spite of the poor recall of risks, respondents with post-primary formal education who affirmed that their concerns are satisfactorily addressed are more likely to recall better (*p* = 0.02).

Formal education is expected to reduce the paternalistic tendencies of individuals who have acquired it. Invitation to ask questions is a recognized approach to increasing understanding [33]. The conviction of patients that their concerns with respect to the disease conditions and planned interventions have been satisfactorily addressed is associated with increased extent of recall of the content of the discussions that addressed those concerns, akin to the findings of van Osch et al. [34]. This finding might be useful in our low socio-economic setting where procedure-specific and multimedia-based consent programs which have been demonstrated to improve information delivery and patient recall [35, 36] are not routine. Every effort must be made to ensure that all the recognized domains of treatment informed consent [37] are addressed up until the concerns of every individual patient are taken care of to the patient’s satisfaction before the signing of the consent form.

## Conclusion

The findings from this study suggest that satisfying consent from the patients’ perspective is associated with better recall of consent information for surgical procedures in low income setting. Therefore, no effort should be spared by the surgeons (and indeed all healthcare giver) in ensuring that the consent information is satisfying to the individual patient from the latter’s viewpoint. Appropriate recall of consent information as an index of understanding by the patients is better among patients whose concerns relating to the disease conditions and treatment decisions are satisfactorily addressed than those whose concerns are not.

## Additional file

Additional file 1: Recall of consent information data set. Description of Data: A data set of the responses obtained from study participants who attempted to recall the content of the consent information received. (PDF 724 kb)

## Abbreviations

AR: Appropriate response
F: Female
Gen: Gender
HEA: Highest level of formal education attained
IR: Inappropriate response
M: Male
NP: No or primary level of formal education
ST: Secondary or tertiary level of formal education
