# Supplementary material for: Informed consent for clinical treatment in low-income setting: evaluating the relationship between satisfying consent and extent of recall of consent information
Source: BMC Med Ethics. 2017 Dec 2;18:69. doi: 10.1186/s12910-017-0227-4 (PMC5712194; doi:10.1186/s12910-017-0227-4)
Supplement: Additional file 1: — Recall of consent information data set. Description of Data: A data set of the responses obtained from study participants who attempted to recall the content of the consent information received. (PDF 724 kb) [file 12910_2017_227_MOESM1_ESM.pdf]

**Recall of consent information study.**

I am an Intern Doctor working with and representing the surgical unit handling your medical condition here in University of Nigeria Teaching Hospital, Ituku-Ozalla, Enugu. We are conducting a study on **"Extent of recall of consent information by patients".**

I would like you to attempt to answer the next few questions to the best of your ability in line with the discussion you had with your doctor a few days ago.

If you do not remember having a discussion along the line of a particular question, kindly say so. If you have forgotten what was discussed, please say so.

Any information you have given is handled confidentially.

Please note that you are not under any compulsion to accept to participate in this study. You will not be exposed to any risk or pain during this interview session as well.

This interview session stops at any point you do not feel comfortable and you indicate that you do not wish to continue. Indicating that the interview session stopped will not affect your continuing treatment.

You are free to ask your questions for further clarification at any point during the interview session.

Kindly indicate that you have given your consent to be interviewed by saying **"YES".**

On the other hand, if you do not wish to be interviewed, please say **"NO".**

Thank you very much.

## ***Patient's comprehension of Informed Consent***

**Section A: Biodata**

Date: ____________________ Folder No: _____________________ PT No: _________________

Age: _______________ Sex: _______________ Marital status: _________________

Highest formal education: [none] [primary] [secondary] [tertiary]

Occupation: [none] [studying] [trading] [farming] [civil/public service] [clergy]

Religious belief: [none] [Christianity] [Islamism] [Traditional] [Others___________________]

Nationality: _______________________________ Tribe: __________________________________

Place of Residence: _____________________________________________________________________

**Section B: This section assesses your understanding of your health condition and planned treatment.**

**Question 1**: May I know your understanding of the diagnosis?

[correct] [incorrect] [forgotten] [uninformed]

**Question 2**: May I know your understanding of the procedure?

[correct] [incorrect] [forgotten] [uninformed]

**Question 3**: May I know your understanding of the risk(s) involved in this planned procedure?

[correct] [incorrect] [forgotten] [uninformed]

Were your concerns/fears about this procedure satisfactorily addressed?

[yes] [no] [unsure]

We appreciate you for your time and co-operation. Thank you so much.

Nnabugwu Ikenna
(Research coordinator)
